# The urban *Triatoma infestans* challenge: integrative insights for vector control and Chagas prevention policies in San Juan, Argentina

**DOI:** 10.1186/s13071-025-07163-6

**Published:** 2026-01-06

**Authors:** Paz Sanchez-Casaccia, Julieta Nattero, Romina V. Piccinali, Anneris Gomez, Marina Ibáñez-Shimabukuro, Mariana Sanmartino, Soledad Ceccarelli, Liliana Salvá, Florencia Cano-Suárez, Sergio Meli, Leonardo M. Díaz-Nieto, Yael Provecho, María del Pilar Fernández, Ana Laura Carbajal-de-la-Fuente

**Affiliations:** 1 Consejo Nacional de Investigaciones Científicas y Técnicas (CONICET), Buenos Aires, Argentina; 2 Centro Nacional de Diagnóstico e Investigación en Endemo-Epidemias, Administración Nacional de Laboratorio e Institutos en Salud (ANLIS) “Dr. Carlos Malbrán”, Buenos Aires, Argentina; 3 Facultad de Ciencias Exactas y Naturales. Departamento de Ecología, Genética y Evolución, Universidad de Buenos Aires, Ciudad Autónoma de Buenos Aires, Argentina; 4 Laboratorio de Eco-Epidemiología, Departamento de Ecología, Genética y Evolución (DEGE), Facultad de Ciencias Exactas y Naturales (FCEyN), Universidad de Buenos Aires (UBA), Buenos Aires, Argentina; 5 Instituto de Ecología, Genética y Evolución de Buenos Aires (IEGEBA), CONICET-Universidad de Buenos Aires, Ciudad Autónoma de Buenos Aires, Argentina; 6 Centro de Estudios Parasitológicos y de Vectores (CEPAVE)/CONICET-CCT-La Plata, Universidad Nacional de La Plata-Asociado a CIC BA, La Plata, Argentina; 7 Grupo ¿De Qué Hablamos Cuando Hablamos de Chagas? (Asociación Civil Hablemos de Chagas), La Plata, Argentina; 8 Consejo Nacional de Investigaciones Científicas y Técnicas, Instituto de Física de Líquidos y Sistemas Biológicos, Grupo de Didáctica de Las Ciencias, La Plata, Buenos Aires Argentina; 9 Programa de Control de Enfermedades de Transmisión Vectorial de San Juan, Ministerio de Salud Pública de San Juan, San Juan, Argentina; 10 Instituto y Museo de Ciencias Naturales, Departamento de Biología/Facultad de Ciencias Exactas, Físicas y Naturales (FCEFyN), Universidad Nacional de San Juan-CONICET, San Juan, Argentina; 11 Dirección de Control de Enfermedades Transmitidas por Vectores, Ministerio de Salud de La Nación, Buenos Aires, Argentina; 12 Paul G. Allen School for Global Health, Washington State University, Pullman, WA USA

**Keywords:** Urban triatomine infestation, Chagas disease, Integrated management

## Abstract

**Background:**

Chagas disease has historically been linked to triatomines and rural areas. However, urban infestations by one of its vectors, *Triatoma infestans*, are increasingly being reported. Urbanization is reshaping vectorial transmission patterns of this disease, creating new collective health challenges. To provide evidence on the eco-epidemiological status of Chagas in the metropolitan region of San Juan, Argentina, this study integrates data collected on biomedical, epidemiological, socioenvironmental, and territorial factors.

**Methods:**

We conducted a cross-sectional analysis of 432 urban houses for infestations by *Triatoma infestans* and infection of these vectors with *Trypanosoma cruzi*, complemented by environmental, sociodemographic, and human practices surveys. Additionally, we carried out information, education, and communication (IEC) activities to engage with and become acquainted with the community. The IEC activities included immersive virtual reality experiences, community dialogue, and educational games in public spaces.

**Results:**

Our study revealed a house infestation prevalence with *T. infestans* of 10% both indoors and in the houses’ outdoor spaces; *T. cruzi* infection was not detectable in any of the insects*.* Wind was identified as an environmental factor associated with house infestation, as was the presence of chicken coops, in addition to the condition of the houses (structural condition, such as cracks and poor plastering, and how the outdoor space of the houses was used, e.g., for the storage of objects that had accumulated over time). A combination of sociodemographic and environmental factors influenced *T. infestans* infestation prevalence. The IEC activities reached over 150 community members and promoted a dialogue about Chagas disease and vector control. The virtual reality and educational games encouraged strong youth engagement, and the media campaign helped raise awareness and visibility of the issue in the region.

**Conclusions:**

The infestation prevalence of *T. infestans* in the urban area of San Juan highlights the need for urban-specific control strategies that differ from those used in rural settings. The key findings of this study, such as chicken coops being infestation hotspots and the importance of wind direction, and the unique urban context (high-density housing, a territorial institutional presence, and community networks), enable us to recognize opportunities for integrated, multi-actor control frameworks that actively involve communities.

**Graphical Abstract:**

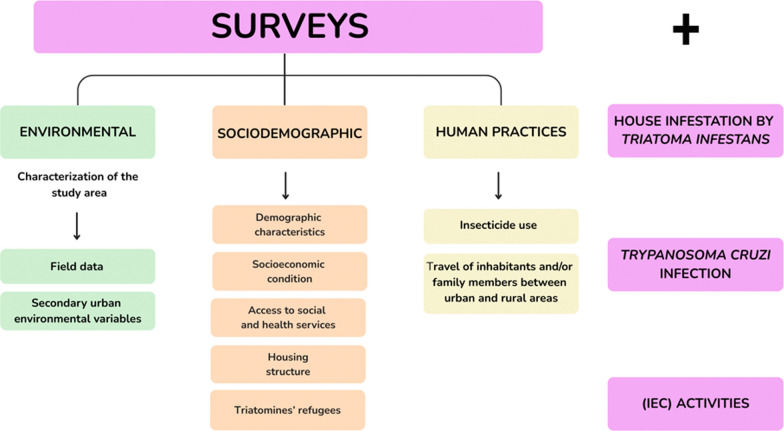

**Supplementary Information:**

The online version contains supplementary material available at 10.1186/s13071-025-07163-6.

## Background

Chagas disease is a complex socioenvironmental health phenomenon, in which various factors—biomedical, epidemiological, sociocultural, environmental and political—converge and interact [1]. It is a multifarious condition affecting an estimated 7 million people worldwide [2]. Following Sanmartino et al. [1], we use the term “Chagas” rather than “Chagas disease” henceforth throughout this text to reflect the multidimensional nature of this disease beyond its biomedical pathology. This broader conceptualization, moving beyond the hegemonic biomedical paradigm, acknowledges the interactions of the etiology, global distribution (driven by human mobility), and multidimensional impacts of Chagas on health and daily life.

On the American continent, the primary transmission route of the parasite *Trypanosoma cruzi* (Chagas, 1909) remains vectorial, via infected triatomine bug feces, while vertical transmission dominates in areas where vectors are rare or absent [2, 3]. Although historically associated with rural housing, triatomine colonization in urban environments, particularly houses, has been increasingly documented across the American continent yet overlooked by vector control agencies [4, 5]. In Argentina, *Triatoma infestans* (Klug, 1834) (Hemiptera: Reduviidae) is the most epidemiologically important vector of *Trypanosoma cruzi* [6, 7]. Reports have confirmed the presence of urban triatomine populations in the metropolitan areas of Catamarca, La Rioja, Mendoza, San Juan, and San Luis in western Argentina [7]. Although, the characteristics used to outline an area as “urban” vary depending on the country and the definitions adopted by international organizations, in general terms, an urban area is characterized by a high population density, developed infrastructure, predominantly non-agricultural land use, access to electric light, sewage and drinking water systems, economic dynamism, physical and design features, and administrative or political recognition [8].

The *T. cruzi* transmission cycle in urban contexts presents unique challenges, partly due to the characteristics, such as a high human population density, high housing density, and differences in the daily practices of residents [9–11]. Urbanization creates conditions that facilitate the spread of vector-borne diseases [12]. Furthermore, vector control strategies for Chagas were originally designed for rural areas and are not always applicable to urban contexts [13]. These limitations highlight the need for government vector control agencies to adapt and implement tools and validate new ones to prevent the emergence of new triatomine foci and the growth of existing ones in an urban context. Recently, as part of efforts to develop these types of strategies, our research team collaborated with provincial and national health authorities to launch a technical document [14].

Despite numerous studies linking urban triatomine infestations to environmental, social, and cultural factors, key aspects such as infestation prevalence, vector infectivity, infected urban hosts, colonized ecotopes, and triatomine adaptation to urban environments remain poorly understood [4, 9, 15–17]. In the metropolitan region of San Juan, western Argentina, the occurrence of *T. infestans* has been linked to housing materials and residents’ behaviors, as well as the presence of domestic animals [18, 19]. It has also anecdotally been associated with the "Zonda" wind, palm trees [19, 20], artificial outdoor lights, and other environmental variables in human-inhabited areas [21], but these associations remain largely unverified, with only a few studies providing limited or indirect evidence for them. Moreover, Chagas research from a social perspective, particularly regarding people’s knowledge and behaviors [22], is still limited and has mostly focused on rural inhabitants, leaving significant gaps in our understanding of how urban residents perceive and respond to this issue.

Given the emerging challenges associated with the urban Chagas, long-term partnerships, strong community engagement, and innovative surveillance strategies are essential for improving vector monitoring and reducing the urban risk of *T. cruzi* transmission as a public health concern [10]. As part of a collaborative inter-institutional consortium set up to deal with urban Chagas, a working group was recently formed, with national members from Argentina and international collaborators from Brazil and Uruguay, including representatives from the Ministry of Health, provincial health authorities, and research groups, with the aim of developing specific approaches to address urban triatomine infestations [7, 23]. These efforts are particularly urgently required in San Juan, which currently represents a unique epidemiological setting. San Juan is the only province in Argentina with documented active vectorial transmission of *T. cruzi* in urban areas in the past 5 years [7]. To gather compelling evidence on the eco-epidemiological status of Chagas in the metropolitan region of San Juan, we carried out an in-depth study addressing vector infestation and *T. cruzi* transmission. The following questions guided our study: (1) What is the prevalence and spatial distribution of *T. infestans* infestation in urban houses? (2) Is there evidence of *T. cruzi* infection in triatomine vectors? (3) Which environmental factors are associated with *T. infestans* infestation? (4) How do sociodemographic and environmental factors and human practices contribute to infestation risk? (5) To what extent do information, education, and communication (IEC) activities create opportunities for knowledge exchange and community involvement in addressing Chagas challenges?

We conducted a cross-sectional analysis of house infestations by *T. infestans* and *T. cruzi* infection of the latter within a well-defined urban area of the Rivadavia department, complemented by surveys of environmental and sociodemographic factors and human practices that could influence the occurrence of *T. infestans*. Additionally, IEC activities were carried out within the local community.

## Methods

### Study area

**Fig. 1A–C:**
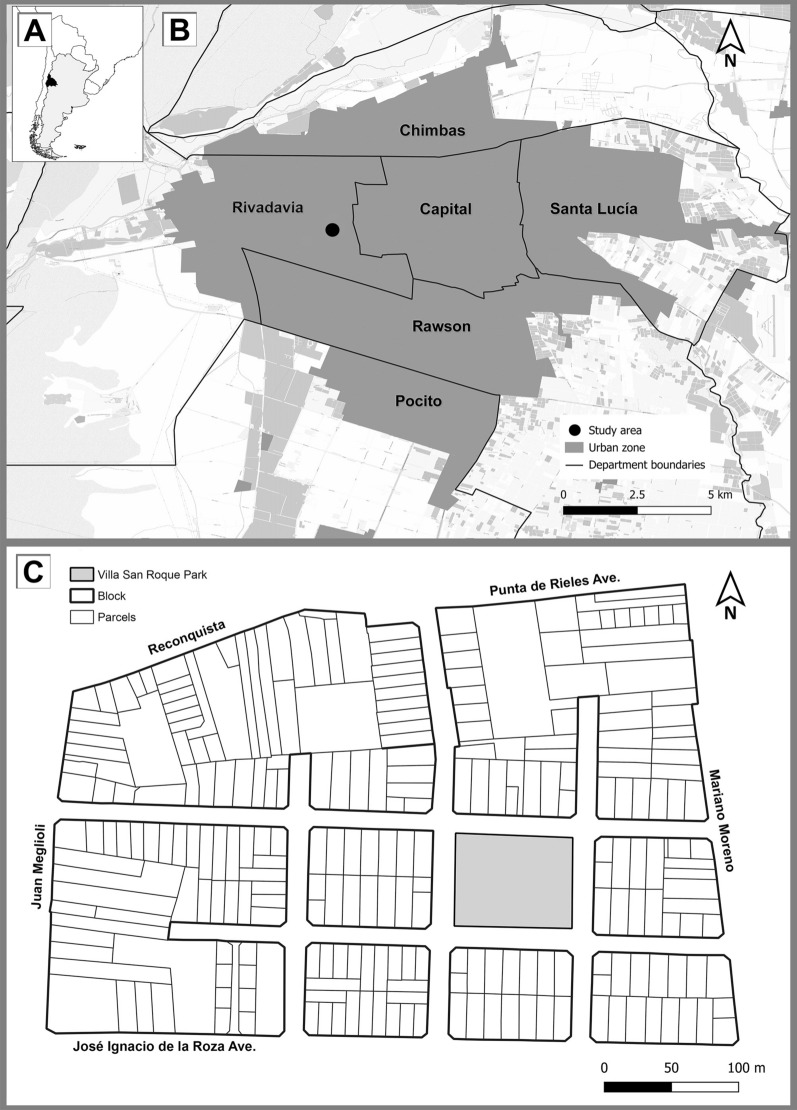
Map showing the location of the study area. **A** Map of South America showing the geographical location of Argentina (grey) and San Juan province (black). **B** San Juan metropolitan region (dark grey) and Villa San Roque (black dot). **C** Neighborhood of Villa San Roque. Maps were generated using open-source geographic information system (GIS) software QGIS 3.22 (QGIS Development Team, https://www.qgis.org/es/site/). Base layers were obtained from publicly available data from OpenStreetMap, cadastral data and the Instituto Geográfico Nacional (https://www.ign.gob.ar/NuestrasActividades/InformacionGeoespacial/CapasSIG)

The metropolitan region of San Juan, also known as “Gran San Juan,” is located in the south-central part of San Juan province, Argentina, and includes the provincial capital (department capital) and neighboring urban areas of the adjacent departments of Chimbas, Rawson, Santa Lucía, Rivadavia, and Pocito (Fig. 1A, B). Gran San Juan has a population of approximately 595,000 inhabitants [24]. Although this area has urban characteristics, it is located within the Monte Desert eco-region [25]. This ecosystem has been transformed by irrigation systems that have created oases within the desert [26]. The arid climate has summer-concentrated precipitation (under 250 mm, and down to 30 mm in some valleys) and an annual mean temperature of 17.9 °C. It falls within the BWk category of the Köppen-Geiger classification, with temperatures ranging from 3.3 °C to 32 °C due to continentality and aridity [27, 28]. One of the main climatic factors influencing the region is the Zonda wind, a dry, warm wind from the eastern Andes, originating in the Pacific anticyclone and modified by the pre-Cordillera cyclone. When this wind reaches Argentina between May and October, adiabatic compression raises its temperature to around 40 °C [29].

The study was conducted during October 2022 in the Villa San Roque neighborhood (31°32′15″S, 68°34′30″W), located in the Rivadavia department (Fig. 1A–C). In 2022, the mean maximum and minimum temperatures were 28 °C and 14 °C, respectively, with a mean relative humidity of 46% [30]. The Rivadavia department shares its western jurisdictional limit with the capital city of San Juan and has approximately 31,900 houses and 102,000 inhabitants [24]. Villa San Roque is located in proximity to areas where *T. infestans* was reported by the San Juan Vector Control Program, but, to date, have no history of entomological evaluations or insecticide spraying conducted by the vector program. The study area covers approximately 18 ha and consists of eight blocks comprising 255 parcels and a total of 432 houses (cadastral data) (Fig. 1C). The parcels vary in area and have defined boundaries. The houses on each parcel are mainly single story and have backyards that exhibiting characteristics typical of urban areas; there are also multi-story buildings (e.g., blocks of flats or condominiums), where the minimal distance between indoor and outdoor spaces (yards) results in a lack of truly peridomestic structures (e.g., goat corrals). There is also a commercial area, while a centrally situated park, which is surrounded mainly by residential buildings, serves as the main green space.

### Study design, entomological surveys,* Trypanosoma cruzi* detection, and IEC activities

This study was conceived as part of rapid intervention work coordinated together with national and provincial health agencies to control vector infestation and *T. cruzi* transmission. In October 2022, six work teams visited all 432 houses of the study area on a daily basis over a period of 3 consecutive weeks. Each team, comprising three to four members, conducted entomological surveys along with assessments of environmental and sociodemographic factors and human practices. The entomological survey was conducted using timed manual searches by specialized personnel from the San Juan Vector Control Program and from the National Vector Control Program of the ministry of Health. All accessible houses were inspected for 15 min both indoors and outdoors, using the person-hour method, for triatomines. A house was considered infested when at least one live specimen of *T. infestans* was found (excluding eggs). Each infested house and the neighboring houses that granted access to our teams were also evaluated and sprayed with an insecticide (beta-cypermethrin spray, 5% concentration of Sipertrin; Chemotécnica, Argentina).

The collected specimens of *T. infestans* were stored in plastic bags together with information on their capture. At the laboratory, all of the specimens were taxonomically identified following Lent and Wygodzinsky [31], separated by stage and sex, and preserved in individual tubes at −20 °C for *T. cruzi* infection analysis. Fourth- and fifth-instar nymphs (*n* = 160) and adult male and female *T. infestans* (*n* = 103), collected both indoors and from the outdoor areas of the houses, were dissected. In cases where an infested house did not have at least one specimen of these stages, third-instar nymphs (*n *= 8) were also included in the sampling. From the total dissected triatomines, pools averaging four specimens each were formed (*n* = 77).

After dissection, the rectal ampulla was extracted and suspended in 50 µL of phosphate buffered saline to facilitate homogenization. The pools were assembled according to the house of origin and developmental stage. DNA extraction was performed using PURO Genomic DNA (Productos Bio-Lógicos, Argentina) according to the manufacturer’s protocol, with slight modifications [32, 33]. The 121/122 primers were used for molecular detection of *T. cruzi* through the amplification of 330 base pairs of kinetoplast DNA, as described by Wincker et al. [34], under the following thermocycling profile: initial denaturation at 94 °C for 5 min; 30 cycles of amplification (94 °C for 45 s, 55 °C for 30 s, 72 °C for 25 s), followed by a final extension step at 72 °C for 3 min. Control DNA (*T. cruzi* extracted as positive) and ultrapure water were used as the non-template control. All polymerase chain reactions were conducted using a Trident 960 Thermal Cycler (Heal Force, Shanghai, China) in a 12.50-µl reaction mixture containing 6.25 µl of PB-L Master Mix (PB-L Productos Bio-Lógicos, Quilmes, Argentina), 0.4 μM of each forward and reverse primer and 2 μL of the template DNA. The amplification products were visualized under ultraviolet light after electrophoresis on an agarose gel (1.5%) and staining with GelRed.

As part of the study, we conducted IEC activities in partnership with the San Juan Vector Control Program by combining community knowledge-exchange initiatives (e.g., screenings of the immersive virtual reality documentary* El Viaje del Tripanosoma* [35], participatory discussions, and educational games) held in Villa San Roque Park, and made information on Chagas publicly visible through media channels [36]. This approach prioritized dialogic engagement, increased access to early diagnosis, and fostered a collective understanding of Chagas beyond biomedical frameworks.

### Environmental, sociodemographic and human practices surveys

The survey on environmental and sociodemographic factors and human practices was conducted by researchers and senior biology students from the National University of San Juan, who participated voluntarily as part of their academic training. All members of these work groups received prior coaching about the type of work to be performed and the corresponding methodology.

The survey was structured around three key components: environmental and sociodemographic factors, and human practices. The environmental component focused on characterizing the study area, including its spatial aspects, through the integration of field data with secondary urban environmental variables. The following data were collected: (1) house locations, which were validated using freely available satellite images and manually georeferenced by assigning coordinates along the street line; for parcels with multiple houses, coordinates were placed approximately within the parcel to better reflect their spatial distribution and avoid misplacement. (2) The predominant vegetation type in public spaces, according to Martínez Carretero [37]. (3) The locations of palms within property limits. (4) Wind patterns (direction and speed). (5) Locations of streetlamps. (5) Data on urban green spaces.

Wind and streetlamp location data were included to assess their potential influence on *T. infestans* dispersal, whereas the predominant vegetation type and location of palms served to characterize the surrounding vegetation context. Data from the urban green spaces map were used to evaluate green space availability. Wind patterns were recorded by a Davis Vantage Pro2 station installed near the study site, within the campus of the National University of San Juan [30]. Georeferenced public lighting data were collected through field mapping of streetlamps and were manually aligned with street lines. Urban green space data were obtained from Ju et al. [38].

Sociodemographic data were collected as the second component, and data on human practices as the third. A questionnaire with closed and semi-closed questions, answered by an adult resident of each house, was used for both of these components. Sociodemographic data included the participant’s age, gender, place of birth, area of origin, education level, household occupation, whether they were a recipient of a government benefit, received a pension, and/or were enrolled in a health plan. Housing-related data covered wall and roof construction materials, wall plastering and roof plastering, house property status, the presence of accumulated objects in the yard, water tanks, pigeons, rats and domestic animals. Human practices were limited to insecticide use and travel of inhabitants and/or family members between urban and rural areas. Additional variables complementary to human practices and perceptions related to Chagas were also recorded; their analysis will be presented in a forthcoming paper currently in preparation.

### Data analysis

The prevalence of house infestations was calculated as the number of infested houses divided by the total number of evaluated houses. Houses that were closed up or uninhabited, or where access to the team was denied at the time of the survey, were excluded. Relative bug abundance was determined by the number of live specimens of *T. infestans* collected by using the person-hour method, with a sampling period of 15 min [13]. To analyze key environmental and sociodemographic factors and human practices associated with *T. infestans* infestation, we examined predictors of household infestation based on the collected data. A combination of descriptive statistics, bivariate analyses, and multivariate logistic regression models was used to assess these associations and generate an integrated model of infestation risk. Categorical variables are presented as frequencies and percentages, while continuous variables are summarized as medians and interquartile ranges (IQRs). For a detailed summary of all of the variables (original and transformed) and statistical information, see Additional file 1.

### Data preprocessing for model development

For the preprocessing of data used in building the risk infestation model, two aspects were considered: the creation of new composite variables from the sociodemographic data (namely house type, socioeconomic status, and animal hosts); and incorporation of secondary urban environmental variables to characterize potential spatial drivers of *T. infestans* dispersal by generating household-level variables (e.g., the number of lampposts or infested houses within 50 m). The detailed code for data preprocessing, along with its rationale, is available in the Zenodo repository [39] and summarized below.

To construct the composite sociodemographic variables, we applied multiple correspondence analysis (MCA) for categorical data (e.g., construction materials, education, income source), principal component analysis (PCA) when numeric variables were present (e.g., animal host counts), or factor analysis of mixed data (FAMD) when both categorical and continuous variables were present. These multivariate techniques allowed us to reduce complex, multidimensional data into a smaller number of orthogonal dimensions that capture the dominant patterns of variation. Hierarchical cluster analysis was then applied to the first few dimensions retained from MCA, PCA or FAMD to classify households into distinct groups. This approach offers a major advantage over including each original variable individually in the model. By reducing dimensionality and avoiding multicollinearity, this method helps to stabilize model estimates and improve interpretability while preserving key structural information about housing, socioeconomic conditions, and domestic animal presence.

First, a housing type variable was created based on wall and roof construction materials and their characteristics. Walls were categorized as adobe (sun-dried earthen building materials composed of clay-rich soil, water, and natural fibers), brick, or mixed materials, with mixed materials divided into walls that included adobe (mixed-1) and those without adobe (mixed-2) because adobe was the most common mixed material used in wall construction. Roofs were classified as concrete, wood, mixed materials (wood combined with roof tiles, concrete, or slabs), or other materials that were found in lesser quantities (e.g., roof tiles, sheet metal, slabs). Wall and roof plastering were recorded. The first five dimensions of the MCA accounted for ~ 77% of the variance and were retained for the subsequent hierarchical cluster analysis. The three resulting clusters were visualized and interpreted based on the distribution of construction characteristics across households, and were used to define the composite house type variable for subsequent analyses.

A similar approach was used to construct a composite variable representing household socioeconomic status. An MCA was performed on categorical variables including the highest education level of household members (primary, secondary, tertiary/non-university, university graduate, postgraduate) and household income type (e.g., employer, employee, self-employed, domestic work, unemployed, pension recipient, government benefit recipient, or enrolled in health plans). The first four MCA dimensions, which explained approximately 60% of the total variance, were retained for hierarchical clustering. The resulting three clusters were visualized and interpreted based on the distribution of education and income profiles, and were used to define the composite socioeconomic status variable for subsequent analyses.

Finally, the need for a new summary variable for animal hosts was examined. As an initial step, a Spearman correlation matrix was constructed for the number of animal hosts (dogs, cats, chickens, and other species). A significant positive correlation was observed between the numbers of dogs and cats. A PCA was conducted for the numeric variables, while a FAMD was performed to incorporate both numeric and categorical variables (specifically the presence of rats). The presence of pigeons was excluded, as none of the infested houses contained them. Between the two analyses, the PCA yielded slightly better results, with the first dimension distinguishing houses with a greater total number of animal hosts, while the second dimension distinguished those in particular that had a higher number of chickens and other species of animal hosts. Given that there were four variables, the first two dimensions collectively explain approximately 50% of the variance. Consequently, based on these results and the significant correlation between the numbers of dogs and cats, the selected animal host variables for the infestation models were the number of dogs and the number of chickens, with chicken presence ultimately inferred by the presence of chicken coops. However, consideration was also given to using the first dimension of the PCA as an alternative to the total number of animal hosts in the model. All of the analyses were conducted in the R environment [40] using the FactoMineR and factoextra packages for MCA, PCA, and FAMD analyses [41, 42].

To generate household-level urban environmental variables, wind patterns were characterized by average speed (kilometers per hour) and frequency across all 16 directions of the compass. The northwest exhibited the highest wind speeds, while the south showed the highest frequency of occurrence. For each study household, we quantified the number of infested houses located upwind (i.e., preceding the focal house along the northwest and south wind directions) within a 10 m-wide directional corridor and falling within 50-m and 100-m circular buffers around the household, capturing the number of infested houses upwind and within range. Additionally, the distance to the nearest streetlamp and the number of streetlamps within 50-m and 100-m buffers around each household were assessed. The percentage of green space within these buffers was also included in the analysis. Although the proximity of palms was considered based on our survey data, this variable was ultimately excluded, as none of the infested houses had them. All spatial preprocessing was conducted using the QGIS software version 3.22 [43].

### Data analysis of domestic infestation

We first conducted bivariate analysis of domestic infestation using Fisher’s exact test between categorical explanatory variables and infestation status (response variable), while the non-parametric Wilcox test was performed for each numerical variable. For multivariate analysis, the associations between the explanatory variables and the response variable were tested through multiple logistic regressions. Firth penalized logistic regression models were conducted using the R package logistf, to address small-sample bias and separation issues that may arise when the outcome is rare (less than 10% of observations) and sample sizes are relatively small [44]. Three regression models were developed and tested, competing against each other and ranked based on Akaike’s information criterion (AIC) adjusted for small sample sizes (AICc). Model averaging was performed, incorporating those models with AICc values less than 2 relative to the best model. Potential multicollinearity among the explanatory variables was assessed using the variance inflation factor. We report the results as odds ratios (ORs) with 95% confidence intervals (CIs). The detailed code for data analysis and the infestation model development is available from the Zenodo repository [39] and summarized below.

The first model (M1) examined the house infestation in relation to sociodemographic variables, testing various combinations of the selected variables. Since no significant association was found between socioeconomic clusters and insecticide use (*p* > 0.05), the latter was excluded from the model. There were three versions of M1: M1a included house type (1–3), presence of chicken coops (yes/no), accumulation of objects (yes/no), and the number of dogs; in M1b the number of dogs was replaced with the first PCA dimension (PC1) which corresponded to the host composite variable and represented non-human animal hosts (including dogs, cats, chickens, and other species of animal); and in M1c house type was replaced with wall plastering (yes/no) and roof plastering (yes/no). The second model (M2) examined house infestation in relation to spatial variables (urban environmental variables), including distance to the nearest streetlamp, the number of infested houses to the south within 50-m and 100-m buffers, and the distance to the nearest infested house. Based on bivariate analyses and due to multicollinearity (variance inflation factor > 10) between the number of infested houses to the south within 50-m and 100-m buffers and the distance to the nearest infested house, the final model retained only the number of infested houses to the south within a 100-m buffer and distance to the nearest streetlamp. The third model (M3) integrated variables from the first and second models for a comprehensive analysis in a combined model. The combined model included the presence of chicken coops, the number of infested houses to the south within a 100-m buffer, wall plastering, and roof plastering.

## Results

### Entomological survey and* Trypanosoma cruzi* infection

**Fig. 2:**
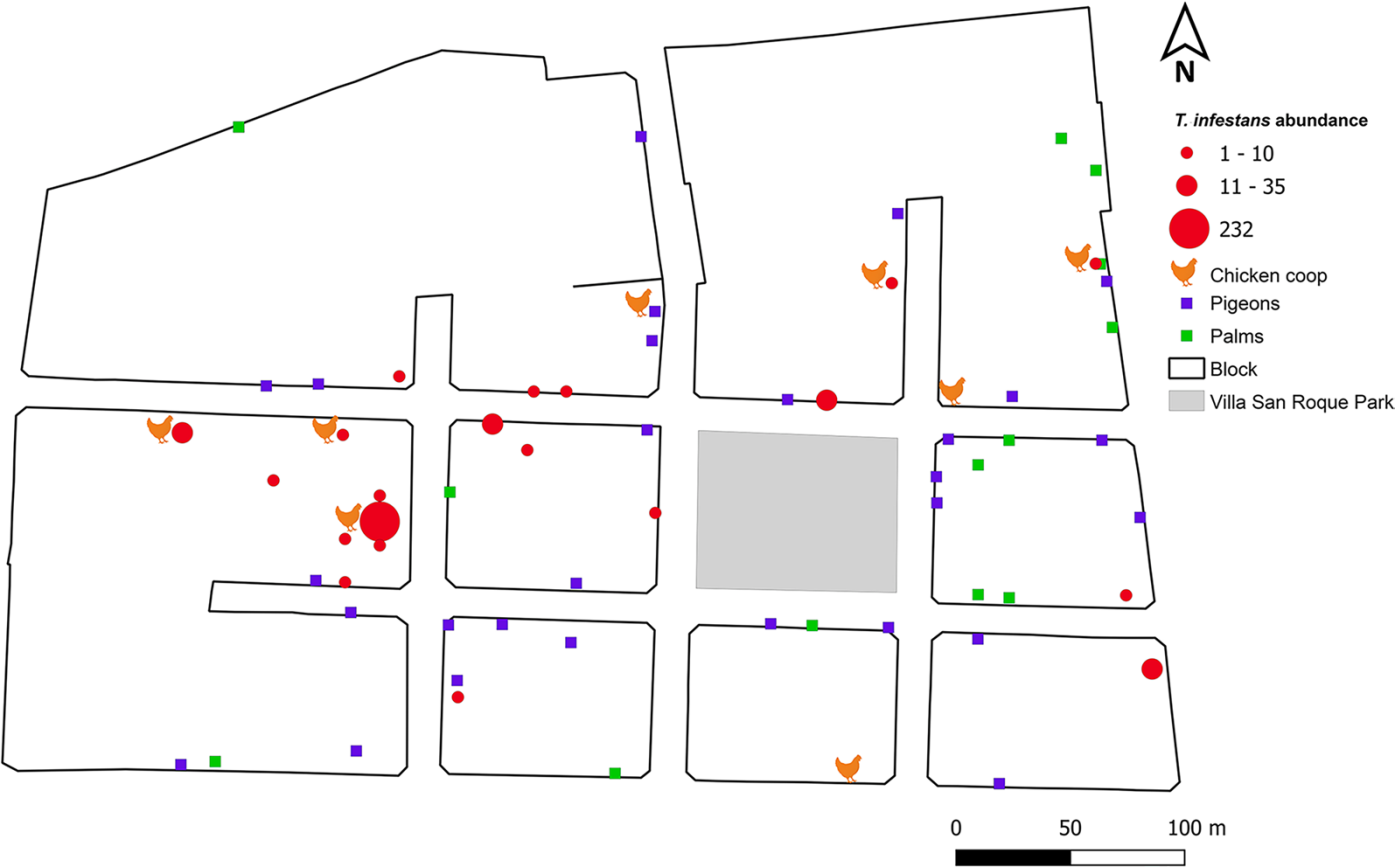
Map showing the spatial distribution and domestic abundance of urban *Triatoma infestans* and locations of houses with pigeons and palms in the neighborhood of Villa San Roque, San Juan, Argentina

**Fig. 3:**
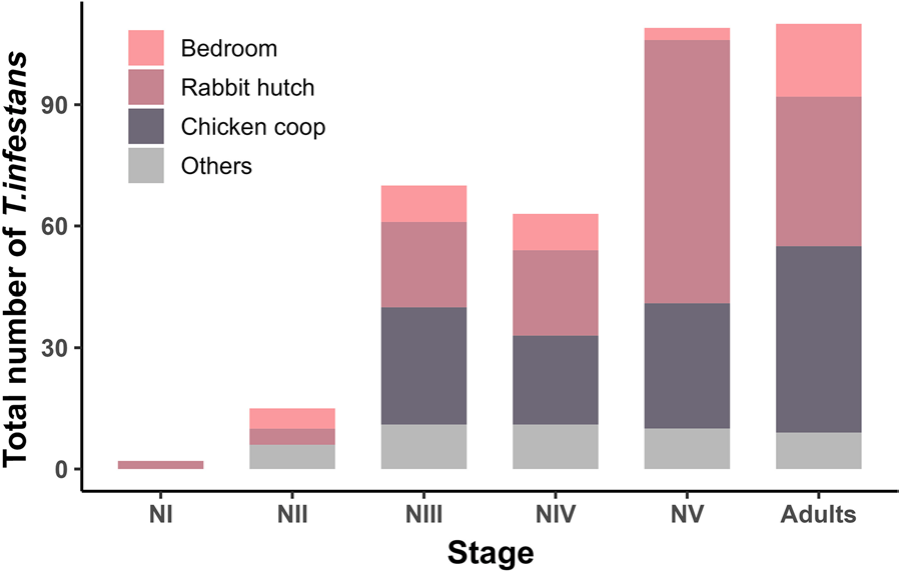
Population structure of *Triatoma infestans* captured within the eco-epidemiological baseline period (October 2022), considering the different capture sites in the infested houses (*n* = 20) in Villa San Roque, San Juan, Argentina.* NI* First nymphal instar,* NII* second nymphal instar, *NIII* third nymphal instar, *NIV* fourth nymphal instar, *NV* fifth nymphal instar

Out of the 432 recorded houses, 201 (47%) were successfully inspected and evaluated for *T. infestans*. The remaining houses were excluded for the following reasons: 166 were closed up at the time of the visit (38%), access was denied for 38 of them (9%), and 27 were unoccupied (6%). All the collected triatomines were identified as *T. infestans*. At least one bug was found in 20 inspected houses, which were located in seven of the eight blocks. This yielded a house infestation prevalence of 10% across all the study houses. Infestations were equally distributed between indoor (50%, *n* = 10/20) and outdoor (50%, *n *= 10/20) areas of the houses (Fig. 2). A total of 369 specimens of *T. infestans* were collected, including adults (*n *= 110) and nymphs (*n *= 259). The median abundance of triatomines per house was two specimens (IQR = 1.75–6.75), with the most frequently captured stages/sex being fifth-stage nymphs (30%, *n* = 109/369), males (15.4%, *n* = 57/369) and females (14.4%, *n* = 53/369) (Fig. 3). One infested household exhibited a markedly higher abundance of triatomines (*n* = 232; 63% of the total *T. infestans* collected), which were found in a chicken coop and a rabbit hutch in the backyard. In contrast, the remaining infested houses showed relatively low densities, ranging from 1 to 35 triatomines. No adult or nymphal triatomine bugs were found infected with *T. cruzi*.

### Environmental, sociodemographic and human practices surveys

The environmental survey recorded ornamental tree species, such as *Morus nigra*, *Platanus hispanica*, *Schinus areira*, *Tecoma stans*, and *Populus alba*. Additionally, palms (family Arecaceae) were present around ca. 7% of the houses (*n* = 13) (Fig. 2). The results derived from the bivariate analyses of urban environmental variables are presented in the “Bivariate analyses” section below.

**Fig. 4:**
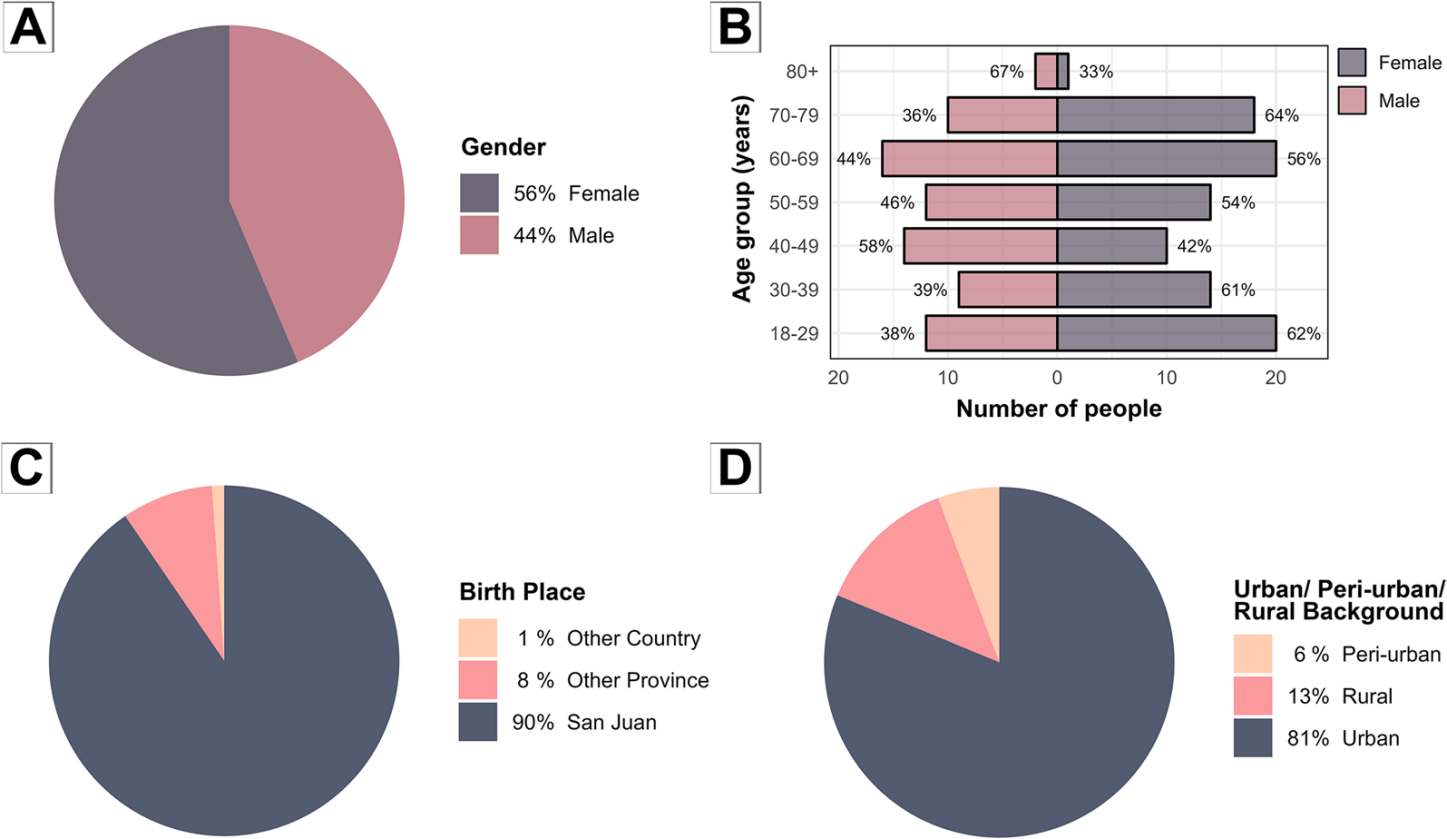
Characteristics of the participants in the sociodemographic survey (*n* = 179) of Villa San Roque, San Juan, Argentina. October 2022: **A** declared gender, **B** age distribution by gender, **C** place of birth, and **D** area of origin

The sociodemographic survey indicated high participation, with 89% (*n* = 179/201) of residents of inspected houses answering the questionnaires. All respondents provided their gender identity, with all responses falling within the categories of men or women. Participation was similar between the genders, although women represented a slightly higher proportion (56%) than men (Fig. 4A). The median age for women was 54 years (IQR = 32–65), comparable to that of men, who had a median age of 50 years (IQR = 36–66), as also shown in the age group distribution (Fig. 4B). Among the particpaants, the vast majority (90%) indicated having being born in the province of San Juan (Fig. 4C), with 81% from urban areas (Fig. 4D). All of the participants had completed at least one level of education, with almost half of the participants having completed secondary education, followed by those who had completed either tertiary non-university education or university degrees, with postgraduate studies being the least common (Table 1). The median number of people per house was three (IQR = 2–4.5). The majority of the participants (67%, *n* = 120/179) owned their houses (median years of residency = 30; IQR = 15–45). Of the remainder, 19% (*n* = 34/179) reported renting their houses (median years of residency = 5; IQR = 1–7), and 8% (*n* = 15/179) described other living arrangements, such as residing in a relative’s house (median years of residency = 20; IQR = 7.5–40). Regarding occupational practices and household incomes, half of the households reported at least one member being a pension recipient (Table 1). Likewise, almost half of the households reported that at least one of them was an employee and a third reported that at least one of the household was self-employed. A government benefit recipient was only reported in 20% of the households, while the majority of the residents (71%) were enrolled in a health insurance plan (Table 1).

Regarding the construction materials of the houses, the main wall materials were bricks (60%), followed by adobe (24%), and mixed materials (16%). For roofing, the most common material was wood (cane or timber) (58%), followed by concrete (24%), and mixed or other less common materials (18%). This pattern was observed in the majority of the studied blocks (five out of eight). Of these structures, approximately 89% of the walls and 46% of the roofs were plastered. Among the households surveyed, the majority (72%, *n* = 128/179) reported owning animals. Dogs were the most common domestic animals, followed by cats and chickens (Table 2). Other species documented included mammalian species (e.g., rabbits, guinea pigs, one horse), a reptile (turtle), and birds (e.g., parrots, canaries) (Table 2). Additionally, 9% (*n* = 34/179) of houses had pigeons or pigeon nests, and 54% (*n* = 96/179) of participants reported occasionally observing rodents in their house.

**Table 1:** Sociodemographic characterization of residents in the households inspected for Chagas in Villa San Roque, San Juan, Argentina, October 2022

Education level	Females, *n* (%)	Males, *n* (%)
Primary	24 (24)	13 (17)
Secondary	41 (41)	37 (47)
Tertiary/non-university	16 (16)	7 (9)
Graduate	8 (8)	15 (19)
Postgraduate	4 (4)	1 (1)
Missing data	8 (8)	5 (6)

Householder occupation and income type	*n* (%)
Employer	11 (7)
Employee	75 (47)
Self-employed	56 (35)
Domestic work	17 (11)
Unemployed	52 (32)
Pension recipient	82 (52)
Government benefit recipient	27 (20)
Health plan enrolled	112 (71)

**Table 2:** Bivariate analyses: *Triatoma infestans* infestations of houses and domestic animals in Villa San Roque, San Juan, Argentina. October 2022

Variable	Number of houses where the animals were present	House infestation status		*p*
		% Not infested (*n*)	% Infested (*n*)	
Dogs	114	88.6 (101)	11.4 (13)	0.23
Cats	51	88.2 (45)	11.8 (6)	0.48
Chickens	8	37.5 (3)	62.5 (5)	< 0.001***
Other species	26	80.8 (21)	19.2 (5)	0.09

*** p ≤ 0.001 (Wilcoxon test* p*-values for comparison of *T. infestans* infestation status by animal presence)

Regarding the human practices survey, three-quarters of the participants (75%, *n* = 135/179) stated that some type of insecticide or pesticide had been applied in their houses in the past 6 months. The most frequently used products were over-the-counter insecticides (53%, *n* = 58/109), followed by those intended for professional use (10%, *n* = 11/109), and others (6%, *n* = 6/109). Some of the participants (31%, *n* = 34/109) reported not knowing which product they had used. The most cited reason for the use of insecticides was the presence of insects such as cockroaches or mosquitoes. Additionally, 46% of households reported traveling to or receiving visitors from rural areas.

### Bivariate analyses

The bivariate analyses of sociodemographic and human practices variables revealed some significant associations with house infestation by *T. infestans*. Infested houses were more likely to have unplastered walls (30.8% versus 8.9% in non-infested houses, p < 0.05) and unplastered roofs (80.0% versus 50.0%, *p* < 0.05) (Table 3). The presence of accumulated objects in the yard was also significantly higher in infested houses (87.5% versus 57.7%, *p* < 0.05). Notably, chicken coops were strongly associated with infestation (27.8% of infested houses had chicken coops versus 1.9% of non-infested houses, *p* < 0.001) (Table 3, Fig. 2).

**Table 3:** Bivariate analyses: *Triatoma infestans* house infestation and spatial variables in Villa San Roque, San Juan, Argentina. October 2022

Variable	*p*
Distance (m) to	
Nearest infested house	0.04*
Nearest streetlamp	0.04*
Nearest infested house (NW)	0.51
Nearest infested house (S)	0.75
Number of streetlamps	
Within 50 m	0.75
Within 100 m	0.02*
Number of infested houses	
Within 50 m	< 0.001***
Within 100 m	< 0.001***
Number of infested houses	
South	
Within 10 m (of the straight line)	0.82
Within 50 m (S)	< 0.001***
Within 100 m (S)	< 0.001***
Northwest	
Within 10 m (of the straight line)	0.81
Within 50 m (NW)	0.51
Within 100 m (NW)	0.35
Percentage of green space	
Within 50 m	0.23
Within 100 m	0.27

*NW* Northwest, *S* south

* *p* ≤ 0.05, *** *p* ≤ 0.001 (Wilcoxon test,* p*-values for spatial variables associated with *T. infestans* infestation)

Analyses of urban environmental variables further revealed significant associations between house infestation by *T. infestans* and proximity to certain environmental features. Proximity to streetlamps and their number within 100 m were significantly associated with infestation (Table 4). The proximity to and the number of infested houses within 50 m and 100 m were also significantly associated with infestation (Table 4). Regarding the effect of wind direction, the number of infested houses within 50 and 100 m upwind in the southern direction (most frequent), but not in the northwest direction (highest wind speed), were significantly associated with infestation (Table 4). The proportion of green space within 50 and 100 m was not significantly associated with infestation by *T. infestans* (Table 4).

**Table 4:** Bivariate analyses: *Triatoma infestans* house infestation and sociodemographic/environmental factors and human practices in Villa San Roque, San Juan, Argentina. October 2022

Variable		House infestation status		*p*
		% Not infested (*n*)	% Infested (*n*)	
Wall construction material				
	Adobe	21.1 (31)	47.1 (8)	0.14
	Brick	61.2 (90)	47.1 (8)	
	Mixed-1	11.6 (17)	5.9 (1)	
	Mixed-2	6.1 (9)	0 (0)	
Wall plastering				
	No	8.9 (11)	30.8 (4)	0.03*
	Yes	91.1 (113)	69.2 (9)	
Roof construction material				
	Concrete	25.0 (36)	17.6 (3)	0.38
	Wood	55.6 (80)	76.5 (13)	
	Mixed	8.3 (12)	5.9 (1)	
	Other	11.1 (16)	0 (0)	
Roof plastering				
	No	50.0 (52)	80.0 (12)	0.04*
	Yes	50.0 (52)	20.0 (3)	
House property status				
	Own	68.8 (106)	93.3 (14)	0.08
	Rented	22.1 (34)	0 (0)	
	Other	9.1 (14)	6.7 (1)	
Accumulated objects in the yard				
	No	42.3 (63)	12.5 (2)	0.02*
	Yes	57.7 (86)	87.5 (14)	
Palms				
	No	91.4 (139)	100.0 (18)	0.36
	Yes	8.6 (13)	0 (0)	
Water tanks				
	No	11.7 (18)	16.7 (3)	0.46
	Yes	88.3 (136)	83.3 (15)	
Presence of pigeons				
	No	78.1 (121)	100.0 (18)	0.02*
	Yes	21.9 (34)	0 (0)	
Chicken coop				
	No	98.1 (155)	72.2 (13)	< 0.001***
	Yes	1.9 (3)	27.8 (5)	
Presence of rats				
	No	45.2 (70)	38.9 (7)	0.80
	Yes	54.8 (85)	61.1 (11)	
Use of insecticides				
	No	20.4 (32)	41.2 (7)	0.06
	Yes	79.6 (125)	58.8 (10)	
Travel to/from rural places				
	No	53.1 (85)	66.7 (12)	0.32
	Yes	46.9 (75)	33.3 (6)	

* *p *≤ 0.05, *** *p* ≤ 0.001 (Fisher’s exact test,* p*-values comparing *T. infestans* infestation status across categories)

### Multivariate analysis

In the first model, where three penalized logistic regression models were fitted (models 1A, 1B, and 1C), model 1C had the lowest AIC. Multi-model inference revealed that only the presence of chicken coops was significantly associated with infestation (OR = 34.05, 95% CI = 2.78–415.84, *p* < 0.01), while other variables included in the model were not significantly associated (Table 5). However, the inclusion of 10 models within a delta AIC of 2—including the null model—suggested that the model was not robust and that a significant amount of the variance remained unexplained by the presence of chicken coops alone (Additional file 2).

**Table 5:** Odds ratio (OR) and confidence interval (CI) for each predictor of urban house infestation by *Triatoma infestans,* Villa San Roque, San Juan, Argentina. October 2022

	Variable	OR (CI 95%)	*p*
Model 1	Intercept	0.09 (0.01—0.60)	0.01**
	PC1	1.08 (0.46—2.48)	0.85
	Roof plastering	0.44 (0.09—2.09)	0.30
	Accumulated objects in the yard	4.43 (0.71—27.43)	0.10
	Wall plastering	0.23 (0.04—1.29)	0.09
	Chicken coop	34.05 (2.78—415.84)	< 0.01**
Model 2	Intercept	0.02 (0.00—0.06)	< 0.001***
	Number of streetlamps within 100 m	1.02 (1.00—1.04)	0.16
	Number of infested houses within 100 m (S)	4.28 (1.51- 2.84)	< 0.001***
Model 3	Intercept	0.09 (0.01—0.48)	< 0.01**
	Number of infested houses within 100 m (S)	3.19 (1.26—8.03)	0.01**
	Roof plastering	0.22 (0.02—2.19)	0.20
	Wall plastering	0.23 (0.03—1.42)	0.11
	Chicken coop	30.64 (3.79—247.25)	< 0.001***

House infestation with *T. infestans* was analyzed through logistic regression, focusing on sociodemographic and spatial variables. The OR, CI 95%, and* p*-values were calculated for each model, with significant results indicated

** p ≤ 0.01, *** p ≤ 0.001

For the second model including the urban environmental variables, the number of infested houses to the south within a 100-m buffer showed a highly significant association with infestation (OR = 4.28, 95% CI = 1.51–2.84, *p* < 0.001). Distance to the nearest streetlamp, however, was not significantly associated with infestation (OR = 1.02, 95% CI = 1.00–1.04, *p* = 0.17) (Table 5).

In the integrated model, although the null model showed the best fit based on AICc values, model averaging identified consistent risk factors, such as the presence of chicken coops, that remained a highly significant risk factor for infestation (OR = 30.64, 95% CI = 3.79–247.25, *p* < 0.001), as well as the number of infested houses to the south within a 100-m buffer (OR = 3.19, 95% CI = 1.26–8.03, *p* < 0.01). Other variables, such as plastered roofs (OR = 0.22, *p* = 0.20) and plastered walls (OR = 0.23, *p *= 0.12), were not statistically significant (Table 5).

### IEC activities

The IEC activities reached over 150 direct observers, including members of the community of Villa San Roque Park, fostering active participation and dialogue about Chagas. The participants reported increased awareness of vector-related risks and control strategies as a result of these activities. The virtual reality experience and educational games were particularly well received, with high engagement among younger participants. Furthermore, the study indicated that the media dissemination campaign contributed to broader public visibility of Chagas in the region.

## Discussion

Our results confirm the occurrence and infestation of *T. infestans* both inside and in the outdoor areas of houses in an urban area of the Rivadavia department, metropolitan region of San Juan, Argentina. The triatomine population structure, which comprised nymphs of all instars, as well as  adults, indicated active colonization of the houses. Notably, neither the nymphs nor adults analyzed were found to be infected with *T. cruzi*. Using a multimodel inference approach, this study provided a deeper understanding of how a combination of sociodemographic factors (e.g., housing materials and their characteristics), human practices (e.g., use of insecticides), and environmental factors (e.g., the relationship with prevailing winds) influence *T. infestans* infestation in urban areas. Multivariate analysis identified wind as a novel risk factor for *T. infestans* infestation of houses, possibly due to its role in  dispersal. Additionally, the presence of chicken coops—traditionally linked with rural environments—was also associated with infestation in this urban setting, highlighting their importance as elements that sustain infestation in urban areas.

In Argentina, urban infestations by *T. infestans* remain a significant challenge for sustainable vector control [4, 14]. Our results are in agreement with previous findings from the Rawson department, another area of Gran San Juan, where 64% of the evaluated blocks had at least one infested house [4]. Although no clear spatial pattern was observed in the Rawson department, in our study we identified a highly infested house in the block with the highest number of infested houses, which possibly indicated a hotspot of infestation. This pattern resembles findings from an urban area in Arequipa, Peru, where infested houses tended to be located near to each other [11].

Triatomines infected with *T. cruzi* have been reported in other areas of the metropolitan region of San Juan, both historically [19, 45] and in recent years [4]. However, the circulation of *T. cruzi* showed variability in transmission dynamics across locations [4, 19, 45]. Moreover, a serological survey of dogs in the Rawson department revealed a 10% *T. cruzi* infection prevalence [4], highlighting the role of domestic animals as potential reservoirs of infection for the vector in the urban transmission cycle and the need for their inclusion in surveillance efforts. A study on Arequipa, Peru, proposed that triatomines facilitate the localized transmission of the parasite within blocks in urban areas, while domestic animals may contribute, though less frequently, to their spread across larger areas, including neighboring blocks or districts [46]. In the present study, although a highly sensitive method (polymerase chain reaction) was used, no *T. cruzi*-infected triatomines were found. In a prior study conducted in another urban area of San Juan [18], the absence of infected triatomines was attributed to the presence of pigeons. Birds are refractory animals for *T. cruzi* infection and constitute a blood source for these insects. In our study, the absence of *T. cruzi* infection in triatomines could be explained by localized transmission dynamics, where the parasite may not have spread beyond a specific area. Another possible explanation is the presence of refractory animals, such as chickens, alongside other non-infected animals, which could sustain local triatomine colonies and may limit their spread. A strong association was found between the presence of chicken coops and *T. infestans* infestation, underscoring the role of these domestic animals as a key risk factor for sustaining *T. infestans* populations. Although chicken coops were few in number in the study area, their presence was remarkable, as their maintenance in urban areas is restricted by local regulations [47]. This finding indicated a gap in regulation enforcement, especially as these urban coops rarely exceed 10 chickens per household and, in some cases, consist of only one or two chickens, likely raised for domestic consumption. These findings emphasize the need for strategies that balance public health goals with local social practices. Control strategies should prioritize improving the management of the chicken coops by encouraging residents to take responsibility for their regular cleaning and maintenance.

In addition to the absence of *T. cruzi*-infected triatomines, there was no evidence of parasite circulation among the human participants of the study. During our IEC activities, volunteers among the community members were offered finger-prick blood sampling on filter paper strips for *T. cruzi* testing (indirect hemagglutination test/enzyme-linked immunosorbent assay) according to Ministry of Health standards [7], for whom no reactive human samples were identified (*n* = 51, data not shown).

Regarding sociodemographic factors, the presence of cracks, gaps, and lack of plastering in construction structures—particularly in walls and roofs—were identified as housing variables that were associated with infestation by *T. infestans* in the bivariate analysis. Similarly, the accumulation of objects around houses was also significantly associated with house infestation, further supporting the idea that cluttered environments provide critical refuges for triatomines. However, these housing characteristics frequently co-occurred with the presence of chicken coops, resulting in potentially redundant information. When considered together in multivariate analyses, these variables lost statistical significance, suggesting that the effect of structural deterioration and clutter may be mediated or confounded by the presence of peridomestic animals. The heterogeneity in wall and roof construction materials across different house types, and household socioeconomic status, showed no significant association with infestation in the study area, indicating that these factors may be less influential in this context and that infestation risks did not differ significantly across households of diverse backgrounds. These findings contrast with those of previous studies undertaken in rural Argentina, where some of these sociodemographic factors showed significant associations with infestation [48, 49].

The absence of an association between house construction materials and the presence of *T. infestans* emphasizes the need for comprehensive interventions that can be effectively applied across varied urban contexts. Such interventions should address both the destigmatization of exposed populations and the use of traditional construction materials like adobe while accounting for urban heterogeneity in housing structures (from permanent earthquake-resistant housing to temporary housing) and their inhabitants [19]. This is particularly relevant in seismically active regions, where adobe has historically been portrayed as a symbol of vulnerability, as exemplified by San Juan’s devastating 1944 earthquake (moment magnitude = 7.0), which nearly destroyed the city and led to its complete reconstruction [50]. As Mandrini et al. [51] emphasize, for them to be effective, strategies must integrate sociocultural perspectives to ensure that interventions aimed at controlling Chagas vectors are socially inclusive. Importantly, the problem lies not in the construction materials themselves but in construction quality, where poor finishes or imperfections (e.g., poorly resolved structural joints, cracks in wall plaster, and discontinuities in roof and ceiling surfaces) create gaps and spaces capable of harboring the vector.

Regarding human practices, our surveys showed widespread use of insecticides in each house, and frequent travel to/from rural areas was commonly reported. As reported in other studies, most individuals apply insecticides independently, usually once a year and outside the framework of an official vector control program, primarily to control nuisance insects rather than triatomines [20]. However, our bivariate analyses revealed no significant association between insecticide application or rural travel and house infestation. This finding regarding insecticide efficacy contrasts with results from other urban areas of San Juan [20], where regular insecticide application, combined with cleaning roofs and balconies, effectively prevented infestation in these highly urbanized areas. The discrepancy between these findings could be due to differences between these houses and those with pigeon nests, or to vector control failures in the study area. Resistance assays on *T. infestans* collected in our study area demonstrated that all of the insects exposed to deltamethrin were susceptible (results not shown). Consistent with these findings is that no resistance has been detected in San Juan Province [52]. These results emphasize the importance of sustained monitoring, as the emergence of insecticide resistance, already reported in other regions of the country [52], could significantly hinder control efforts, especially in densely populated areas where rapid response is essential [14].

In San Juan, palms and pigeons have been associated with triatomine infestation [18, 23], in contraposition to our results*.* Fernández-Maldonado et al. [53] describe a distribution pattern radiating outward from the city center, and found that pigeons were more abundant closer to the center, likely due to their proximity to areas of higher human density and taller buildings (two stories or more), whereas this neighborhood consists mainly of single-story houses, which may explain the lower presence of pigeons. This discrepancy reflects the importance of considering each locality’s unique features.

The models showed that the number of infested houses to the south within a 100-m buffer was significantly associated with infestation. This suggests that wind dispersion is a key factor driving infestation and that the active spread of *T. infestans* is influenced by this environmental factor, potentially facilitating their movement from the outdoor areas of houses to indoors, as well as between houses. Furthermore, wind direction appears to play a significant role in shaping their dispersal patterns. The appearance of *T. infestans* following strong winds, such as the Zonda, in San Juan has been previously documented [18, 20, 45, 54]. However, this phenomenon had not been specifically examined in urban areas until now. Our findings corroborate these earlier observations, further supporting the association between such climatic events and the dispersal of *T. infestans*. In a study on *Triatoma sanguisuga*, a higher proportion of triatomines was collected when the wind blew from the southwest, likely due to the landscape and the presence of sylvatic habitat in that direction, which suggested wind-facilitated dispersal [55]. Some studies have shown a negative correlation between triatomine activity and wind speed [55–57], indicating that while wind direction may facilitate dispersal, higher wind speeds can limit triatomine activity. This is consistent with our finding that the number of infested houses located upwind in the northwest direction (where wind speed is highest) was not significant. In contrast to our wind-related findings, the global multimodel analysis showed weak associations between *T. infestans* infestation and proximity to lampposts, suggesting a limited role for this in explaining infestation patterns in this neighborhood. The attraction of triatomines to artificial light sources has been previously documented in field studies, with triatomine species collected in sylvatic and rural areas [56, 58–60]. In a study by Pacheco-Tucuch et al. [21], rural houses infested by *Triatoma dimidiata* were significantly closer to streetlamps than non-infested houses, with public lights, rather than domestic ones, being associated with infestation. However, our study provides novel insights into the association between light sources and triatomine infestations in an urban context. The abundant artificial lighting in urban areas could reduce the relative attractiveness of individual light sources to triatomines compared to rural settings, where light sources are fewer and more concentrated.

Our study had some limitations. Conducted according to the operational capacity of the provincial and national vector control program, it represents a large-scale effort and highlights the challenges of achieving comprehensive coverage in urban areas. Very few residents refused entry to their homes. Most uninspected houses were closed up or unoccupied, likely because residents were at work during the time period of the visit. This suggests that adjusting the scheduling of control activities to outside working hours could improve access. Furthermore, the study faced three additional methodological limitations related to spatial coverage and sample size. First, despite the high-resolution household-level georeferencing, the spatial analyses may have been affected by the inability to inspect all houses in the neighborhood—approximately half were closed or uninhabited—potentially biasing infestation pattern detection and limiting full spatial coverage. Second, due to the relatively small number of infested houses (*n* = 20), the study faced issues related to statistical power, which constrained the inclusion of multiple predictors and complex interactions in multivariate models. To address potential bias from rare outcomes and data separation, we used Firth’s penalized logistic regression; however, model averaging revealed that no single model was clearly superior, and the best-fitting models had limited explanatory power. Finally, although we incorporated novel spatial predictors, such as wind directionality and public lighting via streetlamp locations, these were derived from secondary data and not validated through entomological movement studies, which limits causal inference. Further research integrating fine-scale movement ecology and larger sample sizes is needed to validate these findings and refine spatial risk models for urban *T. infestans* infestation. Finally, we did not evaluate triatomine blood sources, *T. cruzi* infection in domestic animals, or community perceptions about Chagas; these data are currently being collected and will help clarify infestation and transmission patterns. Additionally, tools to assess the impact of the IEC activities were not applied to this study. While IEC activities were well received and promoted exchange and engagement, the extent to which they fostered sustained community involvement in Chagas-related challenges could not be rigorously assessed.

This study provides novel and valuable insights into the factors associated with *T. infestans* infestation in an urban area of the metropolitan region of San Juan, Argentina. It also serves as a foundational contribution for the Urban Chagas Research Group of Argentina and the Ministry of Health, with observations that support the development of the document* Recommendations for Vector Control of Chagas Disease in Urban Areas* [14]. The presence of chicken coops and the spatial clustering of infested houses, particularly to the south, in relation to prevailing winds, were identified as key determinants. We propose that future interventions should focus on a multi-dimensional and contextualized approach [1], combined with effective management of housing conditions, domestic animals, and improved chemical control strategies (including proper application methods and regular monitoring), while also taking into account local environmental and sociocultural particularities. Additionally, IEC initiatives should be prioritized for a better understanding of communities, to engage their participation and ensure the collective design and successful implementation of prevention and control programs tailored to diverse and complex contexts. A coherent strategy that considers realities on the ground and integrates all aspects of the problem is crucial for achieving sustainable and effective vector control [1]. This integrated framework aligns with earlier proposals for the metropolitan region of San Juan developed by Carrizo Páez et al. [19] and Provecho et al. [4] that highlight the need for context-specific, comprehensive solutions. Inter-institutional collaboration with national and provincial health agencies, research institutes, universities, municipal authorities, civil association and residents is fundamental to achieving this.

Despite severe budget cuts to scientific research in Argentina [61, 62], our group remains committed to studying vectorial urban Chagas. Future research should focus on validating these findings in other settings within metropolitan areas and on exploring additional factors that may influence infestation and infection dynamics, thereby contributing valuable insights to enhance control strategies from an integrated perspective.

## Conclusions

The results of this study contribute to a better understanding of the patterns of *T. infestans* infestation in urban areas of San Juan from a comprehensive perspective, with implications for vector control. The prevalence of infestation in houses was higher (10%) than that reported for other areas of Gran San Juan (7%). This finding reinforces the importance of the work carried out by the Urban Chagas Research Group of Argentina, which emphasizes the need for vector control in urban areas that differsfrom that performed in rural areas. In rural areas, according to current regulations [13], an estimated house infestation prevalence of 5% typically determines whether all houses in a village or district should be treated with insecticide. However, attaining comprehensive coverage for an assessment of infestation or insecticide application is logistically very difficult in densely populated urban settings. Our results confirm active colonization by *T. infestans*, although *T. cruzi* was not detected in any of the vectors analyzed, possibly because their primary blood meals were taken from refractory animals. We identified key environmental factors, including the prevailing wind(s) (associated with southward dispersal), that facilitate dispersion, and urban chicken coops (key refuge sites), together with certain structural conditions of the houses (cracks, poor plastering, accumulation of objects) that help maintain the triatomine populations. The results of this study can be used to strengthen urban vector control guidelines for Chagas, and highlight the importance of interdisciplinary approaches and public policies that take into consideration environmental and sociodemographic factors as well as  human practices in the development of vector control programs.

## Supplementary Information

Additional file 1.

Additional file 2.

## Data Availability

The datasets supporting the conclusions of this study are included in this published article and its supplementary files.
