# Supplementary material for: The urban Triatoma infestans challenge: integrative insights for vector control and Chagas prevention policies in San Juan, Argentina
Source: Parasit Vectors. 2026 Jan 6;19:70. doi: 10.1186/s13071-025-07163-6 (PMC12869957; doi:10.1186/s13071-025-07163-6)
Supplement: Supplementary file 1 — Additional file 1. [file 13071_2025_7163_MOESM1_ESM.docx]

Additional file 1: Summary of environmental, sociodemographic, and human practices variables used in statistical analyses, as part of the baseline ecoepidemiological study conducted in Villa San Roque, San Juan, Argentina, in October 2022. Also indicates where each variable is described in the Methods and Results sections.

| Components | Collection data | Data transformation (Yes /No) | Methods section | Final variable | Analysis performed | Results section |
| --- | --- | --- | --- | --- | --- | --- |
| Environmental | Location of infested houses.  Location of infested houses (south and northwest directions.)  Location of street light posts.  UGS data  Palms | Yes: distance to nearest infested house (m);  circular buffers (50 m / 100 m).  Yes: distance to nearest infested house (m); directional corridor (10 m); circular buffers (50 m / 100 m).  Yes: distance to nearest infested house (m); circular buffers (50 m / 100 m).  Yes: circular buffers (50 m / 100 m).  No | Data pre-processing for model development;  Data analysis of domestic infestation.  Data pre-processing for model development;  Data analysis of domestic infestation.  Data pre-processing for model development; Data analysis of domestic infestation.  Data pre-processing for model development  Data analysis; Data analysis of domestic infestation.  Data analysis | Distance to nearest infested house; Number of infested houses (within range).  Distance to nearest infested house (south and northwest directions); Number of infested houses (upwind and within range).  Distance to nearest street light post; Number of street lights posts (within range).  Percentage of green space (within range)  Used as original | Bivariate analysis; multivariate logistic models.  Bivariate analysis; multivariate logistic models.  Bivariate analysis; multivariate logistic models.  Bivariate analysis.  Descriptive statistics; bivariate analysis. | Table 3  Table 3; Table 5  Table 3; Table 5  Table 3  Table 4; Figure 2 |
| Sociodemographic | Age, gender, birth place, area of origin, education level, number of people, household occupation, government benefit recipient, pension recipient, health plan  enrollment.  Wall construction material, roof construction material, house property status, water tanks, presence of pigeons, chicken coop, presence of rats.  Wall plastering, roof plastering, accumulated objects in the yard, chicken coop.  Wall construction material, roof construction material + wall plastering, roof plastering.  Education level + household income type (household occupation, government benefit recipient, pension recipient, health plan enrollment).  Number of domestic animals (dogs, cats, chickens, other species) | No  No  No  Yes: MCA + Clustering  Yes: MCA + Clustering  No | Data analysis  Data analysis; Data analysis of domestic infestation.  Data analysis; Data pre-processing for model development; Data analysis of domestic infestation.  Data pre-processing for model development; Data analysis of domestic infestation  Data pre-processing for model development; Data analysis of domestic infestation  Data analysis; Data analysis of domestic infestation | Used as original  Used as original.  Used as original  House type (3 groups)  Socioeconomic status (3 groups)  Used as original | Descriptive statistics  Descriptive statistics; bivariate analysis.  Descriptive statistics; bivariate analysis; Multivariate logistic models  Multivariate logistic models  Multivariate logistic models  Bivariate analyses | Table 1; Figure 4  Table 4  Table 4; Table 5  Methods section only (see text)  Methods section only (see text)  Table 2 |
|  | Number of domestic animals (dogs, cats, chickens, other species). | Yes: PCA | Data pre-processing for model development; Data analysis of domestic infestation | Animal host summary variable (PC1) | Multivariate logistic models | Table 5 |
|  |  |  |  |  |  |  |
| Human practices | Use of insecticides; travel to/ from rural places. | No | Data analysis; Data analysis of domestic infestation | Used as original | Bivariate analyses | Table 4 |
|  |  |  |  |  |  |  |
