# Supplementary material for: The urban Triatoma infestans challenge: integrative insights for vector control and Chagas prevention policies in San Juan, Argentina
Source: Parasit Vectors. 2026 Jan 6;19:70. doi: 10.1186/s13071-025-07163-6 (PMC12869957; doi:10.1186/s13071-025-07163-6)
Supplement: Supplementary file 2 — Additional file 2. [file 13071_2025_7163_MOESM2_ESM.docx]

Additional file 2: Top-ranked models from model averaging showing AICc, Delta AIC and AIC weights for model comparison.

| Model variables | AICc | Delta AIC | AIC weights |
| --- | --- | --- | --- |
| PC1 | 56.38 | 0.00 | 0.15 |
| (Null) | 56.42 | 0.04 | 0.14 |
| PC1 + Plastered roofs | 57.80 | 1.41 | 0.07 |
| Plastered roofs | 57.82 | 1.44 | 0.07 |
| Accumulated objects in yard | 57.82 | 1.44 | 0.07 |
| Accumulated objects in yard + PC1  PC1 + Plastered walls  Plastered walls  Accumulated objects in yard + Plastered roofs  Accumulated objects in yard + PC1 + Plastered roofs | 57.87  58.85  58.88  59.29  59.34 | 1.49  2.46  2.49  2.90  2.95 | 0.07  0.04  0.04  0.03  0.03 |
